# Automated capture and transfer of human facial expressions to humanoid robots for realistic patient simulation

**DOI:** 10.3389/frobt.2026.1798227

**Published:** 2026-03-24

**Authors:** Patricia Schwarz, Sebastian Spanknebel, Diana Immel, Rene Hurlemann, Andreas Hein, Sandra Hellmers

**Affiliations:** 1 Assistance Systems and Medical Device Technology, Department for Health Services Research, School of Medicine and Health Sciences, Carl von Ossietzky University of Oldenburg, Oldenburg, Germany; 2 Department of Psychiatry and Psychotherapy, School of Medicine and Health Sciences, Carl von Ossietzky University of Oldenburg, Oldenburg, Germany; 3 Karl Jaspers Clinic, Bad Zwischenahn, Germany

**Keywords:** automatic facial expressiontransfer, humanoid robot, medical education, psychiatric patient simulation, simulated patient

## Abstract

Realistic reproduction of human facial expressions is essential for realistic interactions between humans and humanoid robots. This work presents a data-driven framework for transferring human facial expressions to a humanoid robot and a virtual avatar, aiming to enhance emotional expressiveness and assess its applicability in psychiatric training scenarios. The proposed approach enables cross-domain facial expression mapping while accounting for mechanical constraints of robotic actuation. A user study (n = 40) evaluated emotion recognition across three stimulus categories: human faces (H), unconstrained virtual avatars (A) and humanoid robots with limited facial actuation (R). Participants identified emotions from static images and from dynamic expression sequences, presented with and without speech. Perceived realism and uncanny valley effects were assessed using an eight-item questionnaire rated on a 7-point Likert scale. Results indicate that human-to-robot facial expression transfer is feasible but constrained by mechanical expressivity. Highly expressive emotions such as surprise (H: 87.5%; A: 57.5%; R: 65%) and fear (H: 45%; A: 27.5%; R: 57.5%) achieved moderate recognition rates, whereas subtle emotions such as anger (H: 65%; A: 40%; R: 12.5%) and disgust (H: 60%; A: 10%; R: 22.5%) were poorly recognized on the robot. Dynamic expressions combined with speech significantly improved recognition. These findings demonstrate the feasibility of transferring human facial expressions to humanoid robots while highlighting current limitations of robotic facial actuation. The proposed framework provides a promising basis for emotionally realistic patient simulation and training applications in mental healthcare.

## 1 Introduction

In clinical contexts, successful clinician–patient interactions are strongly associated with improved therapeutic response, improved patient outcomes increased trust and higher patient satisfaction (Catty, 2004; Khullar, 2019; Ruberton et al., 2016). The interaction is based on verbal and nonverbal communication and can vary depending on the clinical context (specialty) or the patient’s condition (cognitive or psychiatric profile). In addition to verbal communication, nonverbal communication includes the exchange of information without speech, in particular through facial expressions and gestures (Hall et al., 2019). Facial expressions convey important information about a patient’s emotional state and wellbeing (Hess et al., 2023). Recognizing and interpreting facial expressions and behavioral symptoms plays a central role, especially in psychiatric patient cases (Lehman et al., 2004; Templier et al., 2015). Training and practicing with such patient cases are therefore a central topic in the education of medical professionals. A study by William et al. emphasizes the central role of nonverbal communication and emotional expression for the realistic training of doctors (Laughey et al., 2018). Professional actors are commonly employed as simulated patients in medical education. However, such programs are limited in their ability to consistently reproduce complex diagnostic and therapeutic scenarios and are associated with substantial costs for recruitment and training (Keiser and Turkelson, 2017). In recent years, humanoid robots have emerged as an alternative platform for medical simulation, enabling highly available, repeatable, and human-like training scenarios. In particular, realistic facial expressions and gestures allow humanoid robots to replicate patient behavior relevant to medical and psychiatric education, supporting the training of diagnostic and communication skills. Several studies suggest that simulation of emotions by robots has a positive effect on learning, see Alghamdi et al. (2021). In a previous study, we investigated the use of a humanoid robot as a simulation patient in medical education. The results of this study suggested that the robot is well suited as an alternative learning technology for patient simulation and was accepted by medical students, see Schwarz et al. (2023).

Precise transmission of human facial expressions would allow humanoid robots to simulate a wide range of clinical conditions and psychosocial states. To avoid time-consuming manual animation, automated methods for capturing and transferring human facial expressions to robotic platforms are required. In the field of robotic emotion expression, it has already been shown that humanoid robots are capable of displaying simple emotional states that can be recognized by humans using mechanical actuators and programmed facial expressions (Breazeal, 2003).

However, realistically reproducing complex and nuanced emotions remains a major challenge, particularly when the facial movements of humanoid robots are constrained by a limited number of actuators and degrees of freedom. It is therefore essential to investigate how observers perceive the realism and emotional authenticity of facial expressions displayed by humanoid robots and evaluate them affectively in order to develop improved technical implementation of facial expressions to increase human acceptance of humanoid robots. To ensure that transferred facial expressions are identifiable by observers, a systematic classification of facial expressions is required. The deformation of facial features (facial expressions) can be associated with the expression of basic emotions. As an application example, the facial expressions of the seven basic human emotions according to Paul Ekman (anger, sadness, disgust, joy, contempt, surprise, and fear) are used to evaluate the developed method. Paul Ekman’s theory of the seven basic emotions is considered an established basis for categorizing and analyzing emotional facial expressions (Ekman, 1992). These basic emotions are the only facial expressions that can be clearly read without context (Ekman and Friesen, 1975). This makes it possible to identify the transferred facial expression by naming the emotion shown. The aim of this study is to develop, apply, and evaluate an automated method for transferring human facial expressions to a humanoid robot and a virtual avatar. The developed method is based on three-dimensional face recognition using the Google MediaPipe framework, the assignment of key points, and the conversion of so-called blend shape coefficients into a set variable to control the available actuators in the face of the humanoid robot. Although considerable progress has been made in recent years in the representation of emotional facial expressions in both humanoid robots and virtual avatars, key challenges remain. In particular, there is a lack of automated, integrated methods that enable the realistic, precise, and robust transfer of complex human facial expressions to technical systems with limited actuation capabilities. Existing approaches often focus either on virtual avatars or on simple emotional representations in robots and rarely consider the entire process chain from the capture of real human facial expressions to their mathematical transformation and physical reproduction. Furthermore, there is a lack of systematic empirical studies investigating the extent to which the transferred emotions are reliably recognized by observers and how different forms of representation (physical robot vs. virtual avatar) affect perception, acceptance, and possible discomfort effects. This research gap directly gives rise to the three central research questions of the work:

The first research question addresses the core technical problem by investigating whether and to what extent human facial expressions can be converted into an actuator vector for a humanoid robot through a purely mathematical transformation. The aim is to determine an optimal mapping function that ensures the highest possible structural correspondence between human facial expressions and robotic reproduction with minimal error. This will make a significant contribution to the development of automated transfer methods that build on current advances in three-dimensional facial recognition and the transfer of human facial expressions to digital avatars (Purps et al., 2021; Alkawaz et al., 2015).

The second research question ties in with the perceptual psychology and communication-related dimension and examines whether dynamic, speech-accompanied facial expressions lead to higher emotion recognition rates than static representations. This tests the extent to which the developed method is not only technically precise but also communicatively effective. This question is central to assessing the practical suitability of the system for medical training scenarios in which nonverbal communication, emotional expressiveness, and multimodal interaction play a crucial role (William et al., 2018; Hall et al., 2019; Hess et al., 2023).

The third research question expands the study to include an acceptance-related perspective by analyzing whether an increasing degree of human likeness potentially causes discomfort or irritation in the sense of the uncanny valley effect. This examines whether the most realistic facial expressions possible are always desirable or whether psychological limits of acceptance must be taken into account, as discussed in current work on human-robot interaction and the real-time transmission of human emotions (Park and Whang, 2022; Spezialetti et al., 2020).

The specific contribution of this work lies in the development and empirical evaluation of a continuous, automated transfer process that integrates modern 3D facial recognition, mathematical transformation, and robotic actuator control. This closes a gap between existing approaches to robotic emotion representation (Breazeal, 2003) and newer methods for realistic emotion transfer to avatars (Purps et al., 2021; Alkawaz et al., 2015). In addition, the study provides quantitative insights into the recognizability, perception, and acceptance of emotional facial expressions in both a physical humanoid robot and a virtual avatar, thereby expanding existing research on the effect of emotional robot simulations on learning processes (Alghamdi et al., 2021; Schwarz et al., 2023). By systematically comparing both forms of representation, the work contributes to deriving design principles for future humanoid simulation systems in medical education and closing the gap between technical feasibility and psychological effectiveness.

## 2 Methods

### 2.1 Overview of the transfer pipeline

The proposed method implements an automated, data-driven pipeline for transferring Paul Ekman’s seven basic human emotions to a humanoid robot and a virtual facial avatar. Facial landmarks and blendshape coefficients are extracted using the MediaPipe facemesh framework, emotion-specific deformations are computed relative to a neutral reference, and these are mathematically mapped onto the robot’s limited facial actuators while accounting for mechanical and representational constraints. An iterative simulation-in-the-loop optimization minimizes the deviation between intended and generated expressions, enabling realistic and reproducible emotional displays on both platforms. Figure 3 provides an overview of the complete processing pipeline. A user study evaluates whether the transferred emotions can be reliably recognized by human observers.

### 2.2 Basic emotions

**FIGURE 1:**
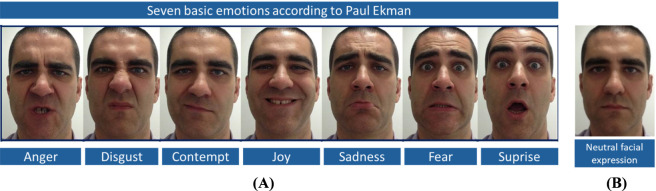
Seven basic emotions (anger, sadness, fear, surprise, disgust, contempt, joy) **(A)** according to Paul Ekman (7 basic emotions, Ekman, 2025) and the neutral facial expression **(B)**.

Validation of the facial expressions displayed by human participants is essential to ensure that the facial expressions shown by the robot or avatar are actually perceived as intended. The work of Ekman and Friesen (Ekman et al., 2002) provides a well-established framework for systematically mapping the movements of individual facial muscles to prototypical emotional expressions. Based on this framework, all seven basic emotions defined by Ekman (anger, sadness, disgust, joy, contempt, surprise, and fear) were selected to investigate the identifiability of the transferred facial expressions (Figure 1). In addition to these emotional expressions, a neutral human facial expression was included as a reference state for the conversion and transfer process.

### 2.3 System components and tools

To ensure a clear understanding of the overall process, we first describe the system components before detailing the mathematical transfer model.

#### 2.3.1 Humanoid robot ameca and virtual robotics simulation

Engineered Arts Ltd. (2022) designed Ameca to closely resemble the human body. The robot has a human-like anatomy with arms, legs, a torso, a head, and a face. The robot is intended to convey emotions, intentions, and establish a deeper connection with people. In addition, the ability to speak and have a human-like voice allows Ameca to communicate in a way that is understandable and familiar to humans. Ameca’s elementary operations can be programmed and tested using the “Virtual Robotics” simulation tool. It provides a virtual environment, allowing the user to create and simulate robot operations in terms of program movements trajectories of rigid robot components and to approximate non-rigid transformations from motor output to flexible skin movements. Of special importance for the simulation-in-the-loop approach used in this paper is the robot’s face, with 13 controllable actuators (facial muscles). These actuators are covered with a flexible coating that can be deformed by the actuators. With Virtual Robot, the Ameca robot can be programmed via a programming interface to define the desired facial expressions and upper body movements. We also use the simulations in Virtual Robot to visualize the avatar and to capture iteratively the actual facial expression.

#### 2.3.2 MediaPipe Facemesh and blendshape representation

**FIGURE 2:**
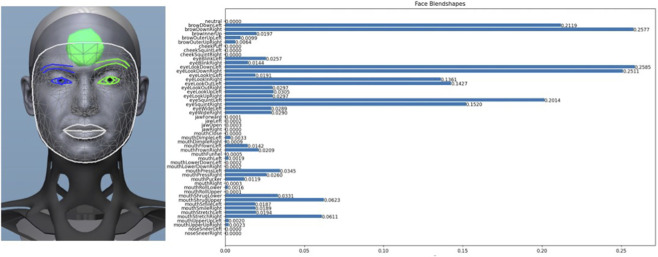
Facemesh on the avatar’s neutral facial expression generated by the tool Virtual Robot (left) and the corresponding 52 blend shape coefficients (right).

The input images from either real humans or the facial expression from the Virtual Robot tool are processed by the MediaPipe Facemesh module, which is responsible for extracting facial features (feature extraction). The main reason for using MediaPipe is the efficiency of its facemesh module (Google MediaPipe, 2025a). For this purpose, a facemesh generated by MediaPipe is used to extract facial expressions from images. The tracking output is 468 3D coordinates Landmarks (x, y, z) for a single mask (1 image or 1 frame in a video). These points describe the geometry of the face and represent semantically relevant regions (such as eyes, nose, mouth, cheeks, forehead, etc.). An example of the application of the facemesh can be seen in Figure 2 (left) in the avatar’s face.

Blendshape coefficient are used for facial recognition and pose recognition. The 468 coordinates serve as the basis for this, and an artificial neural network uses the coordinates to estimate 52 blendshape values (BV). The blendshape values are predefined morphological states (such as mouth smile left = x). Each blendshape coefficient has a value between 0 and 1, which describes how strongly this expression is activated (activation degree of a blendshape, Google MediaPipe, 2025a). Figure 2 (right) shows the corresponding 52 blendshape coefficients for the avatar’s face. They show values between 0 and 1 for the respective positions of the facial expressions.

For this purpose, the neutral facial expression is defined and the weighted differences of the other facial expressions are added to the neutral facial expression. However, the humanoid robot only has 13 controllable actuators in its face that can be used to adjust facial expressions. It is therefore necessary to map the robot facial actuators to the corresponding blenshape values. To do this, the various robot actuators were systematically adjusted within the degrees of freedom and their influence on the blenshape coefficients was determined (Figure 2). This enabled us to assign the corresponding blendshape coefficients to the 13 robot positions (Supplementary Appendix Table 1a: Assignment of blendshape values to the degrees of freedom).

### 2.4 Facial expression representation and mapping concept

**FIGURE 3:**
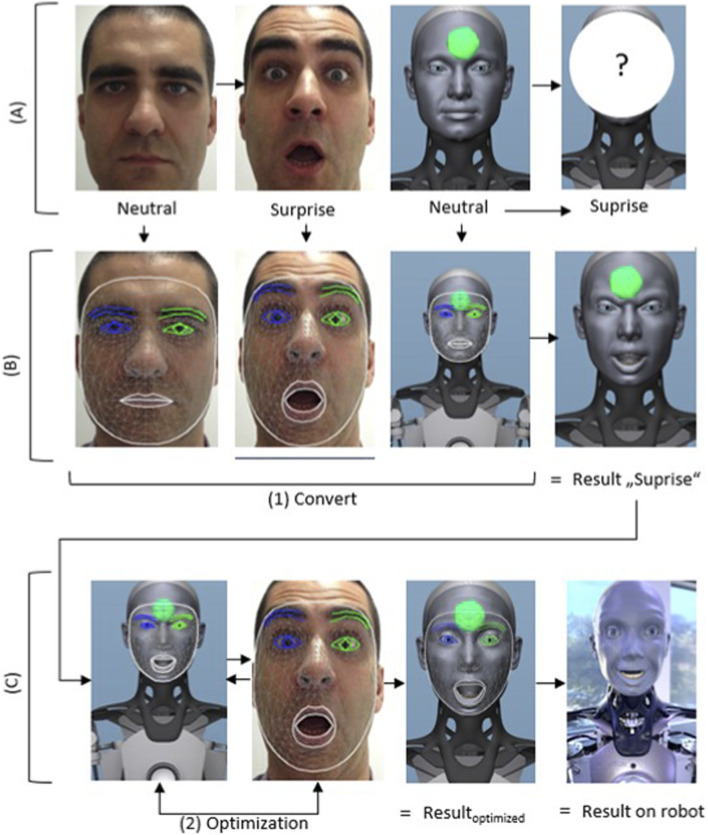
Process for capturing a human facial expression (neutral to surprised), transferring it to a 3D model using face mesh and blend shapes, and then optimizing and implementing it on a robot. **(A)** Input. **(B)** Facemesh + Blendshape values. **(C)** Adaption.

As illustrated in Figure 3, the pipeline consists of four main stages: facial capture, deformation extraction, mathematical mapping, and iterative optimization. The transfer process starts with three inputs: an image of a human face showing a neutral expression, an image of the same human displaying a target emotion (here: surprise), and the neutral facial configuration of the humanoid robot (Figure 3, first line: (A) Input). These inputs (A) define the reference state and the target emotional expression to be transferred.

Both human images (neutral and emotional) are analyzed using the MediaPipe and the blendshape values (BV), (Figure 3, second line: (B) Facemesh and Blendshape values). This step extracts facial keypoints and corresponding blendshape values, which quantitatively describe the deformation of facial regions such as the eyes, eyebrows, mouth, and forehead. By computing the difference between the blend shape values of the emotional and neutral human expressions, a relative representation of the facial deformation associated with the emotion is obtained. The differences are added to the starting position of the avatar positions in the neutral state. After executing the robot positions, a first approximation of the avatar with the facial expression surprise (Result: *surprise*) is created. In a further step, the avatar’s facial expressions are optimized, see Figure 3, third line: (C) Adaption. The avatar’s generated facial expressions are compared with the original human image of the basic emotion, and the robot positions are adjusted iteratively. The final result is an optimized expression of the facial expressions for the selected emotion on the avatar (C: Result_optimized_). The optimized result is finally output to the humanoid robot. Determination of a deviation limit: Human keypoints or blend shape parameters cannot be transferred to the robot face vector without errors due to the mechanical limitations of humanoid actuators.

Therefore, a maximum deviation of ≤ 20% is defined as the tolerance threshold, which takes into account both the structural model incongruity and lies within the threshold accepted by perception psychology, since deviations of up to 15%–25% are generally not perceived as incorrect facial expressions (Marneweck et al., 2013).

The figure shows a schematic representation of a process for capturing and transferring human emotions to a robot. First, a human facial expression (here: neutral to surprised) is recorded (A). Then, using face mesh and blend shape values, the relevant facial features are extracted and transferred to a digital 3D model (B, Convert). In a further step, the parameters are iteratively optimized (Optimization) in order to precisely adjust the target expression (C, Adaptation). The optimized result is then transferred to the robot face “Result on robot).

After outlining the conceptual mapping approach, the following section summarizes the mathematical formulation.

### 2.5 Mathematical formulation of the transfer

The mathematical conversion of blendshape values (BV) into robot actuator positions (RP_j_) was implemented in Python using a simulation-in-the-loop approach based on the *Virtual Robot* environment. From the 52 blendshape coefficients extracted from human facial images using MediaPipe, a subset was systematically assigned to the 13 facial actuators of the humanoid robot (*j = 1…13*). Table 1a (Supplementary Material) shows the individual assignment of the corresponding blend shape values (BV) to the degrees of freedom of the robot (robot positions, RP). The goal of this procedure is to translate human facial expressions into mechanically feasible and optimal actuator movements.

First, emotion-specific facial deformation patterns are computed by calculating the relative differences between neutral and emotional human facial expressions in the blendshape domain. These deformation patterns form the basis for generating initial actuator commands for the robot (Equation 1). The difference *D*_*i,j*_^*x*^ describes the percentage deviation for the x’th iteration of the respective facial expression from the neutral facial expression and thus forms the basis for the transfer to the robot.

$$D_{i,j}^{x}=\frac{{BV}_{i,j}^{H}-{\;BV}_{n,j}^{H}}{{BV}_{n,j}^{H}}\;{\;BV}_{n,j}^{H}\neq 0$$ (1)

Second, the computed deformation patterns are transferred to the neutral facial configuration of the humanoid robot by applying the established blendshape-to-actuator mapping. During this step, all actuator commands are constrained to remain within the robot’s mechanical degrees of freedom to prevent unrealistic movements or actuator overload (Supplementary Equations 2, 3).

A theoretical target facial expression is estimated for the robot in the blendshape domain. The corresponding actuator commands are executed in the simulation environment and the resulting facial expression is re-analyzed using MediaPipe to extract updated blendshape coefficients (Supplementary Equations 4, 5).

Finally, an iterative optimization process is applied in which actuator commands are progressively refined. In each iteration, the deviation between the theoretically achievable target expression and the actual robot-generated expression is computed, and actuator positions are adjusted accordingly. This loop is repeated until the deviation ( $D_{opt,i,j}$ ) falls below a predefined tolerance threshold of 20%, which is consistent with established perceptual limits (Equation 6).

$${\;D}_{opt,i,j}^{x}=\frac{\left|{\;gBV}_{i,j}^{R,}-{BV}_{i,j}^{R,x}\right|}{{\;gBV}_{i,j}^{R,}}\leq 0.2$$ (6)

The complete mathematical formulation and iterative optimization procedure are provided in Supplementary Equations.

### 2.6 User study design

The facial expressions corresponding to Ekman’s seven basic emotions were transferred to both the humanoid robot and the virtual avatar using the proposed pipeline. The optimized result of each emotion was captured as an image. A total of 21 images, each showing seven basic emotions of humans, avatars, and robots, were shown to a group of 40 participants in randomized order. The aim was to identify the respective emotion in the image. In addition to the static images with the basic emotions, dynamic speech sequences were also generated and evaluated in a user study. Here, seven moving sequences were applied to the robot. In each sequence, the robot spoke a few sentences that were intended to convey a basic emotion. Here, too, the emotions in the dynamic sequence were to be identified. The exact procedure is described in Sections 6.1, 6.2. Afterwards, the participants filled out a questionnaire to assess the uncanny valley effect (Section 6.3).

#### 2.6.1 Identification of facial expressions by basic emotion

The task of the study participants was to identify the 7 facial expressions shown by humans, robots, and avatars based on the basic emotion. The facial expressions were presented to the participants as photos. To do this, the participants were shown a total of 21 images of humans/avatars/robots one after the other. The participants received brief instructions on the background and the study procedure. The seven basic emotions were mentioned in advance and briefly explained. The images were presented to them on a large screen in random order. Each image was shown for about 30 s and the participants were asked to decide intuitively. On a questionnaire handed out in advance, the participants marked their assumed basic emotion for the facial expression shown in the image in each round.

#### 2.6.2 Identification of facial expressions in a dynamic scene

The participants were also shown brief, dynamic sequences in which the humanoid robot speaks a few sentences and shows emotions through the use of facial expressions. The sequences were played to the participants in a live setting by the humanoid robot Ameca. A total of 7 sequences with different sentences were presented to the participants, each containing one of the 7 basic emotions (Acting Script). Here, too, the participants were asked to identify the basic emotions in the sequences. The sequences were based on short videos featuring actors (male and female) who performed scenes illustrating the respective emotions. The videos were then transferred to the humanoid robot and was presented to the participants in random order.

#### 2.6.3 Uncanny valley effect

The visual response to robot faces was evaluated using an established 7-point Likert scale with the following items: disgusting, pleasant, eerie, trustworthy, repulsive, creepy, friendly, and likable (Supplementary Material, Questionnaire 6a). Participants rated the eight characteristics on scales from 1 to 7 (Palomäki et al., 2018).

#### 2.6.4 Participants

40 participants took part in the study. Table 1 shows the distribution of age, profession, work experience and gender. To participate, you must be at least 18 years old and consent to participate in the study. No specific professional background was expected. The Ethics Committee of the University of Oldenburg has approved the study (ethics vote available: Drs.EK/2025/029-01).

**TABLE 1:** Characteristics of Participants, n = 40.

Characteristics	Participants
Age (years)	
Age (years), mean (SD)	29.5 (9.7)
Sex, n (%)	
Male	13 (27.5)
Female	27 (72.5)
Profession, n (%)	
Medical student	1 (2.5)
Researcher	3 (7.5)
Teacher	1 (2.5)
Nurse	35 (87.5)
Profession in years, n (%)	
Profession in years, mean (SD)	3.9 (3.9)

## 3 Results

### 3.1 Identification of basic emotions

**FIGURE 4:**
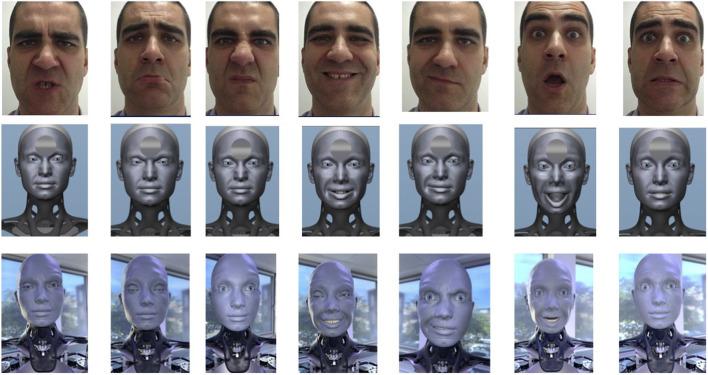
Optimized results of transferring the seven basic human emotions to avatar and robot (from left to right: anger, sadness, disgust, joy, contempt, surprise, and fear).

Figure 4 below shows the result of automatically transferring facial expressions for each of the seven basic human emotions to the avatar and the robot.

**FIGURE 5:**
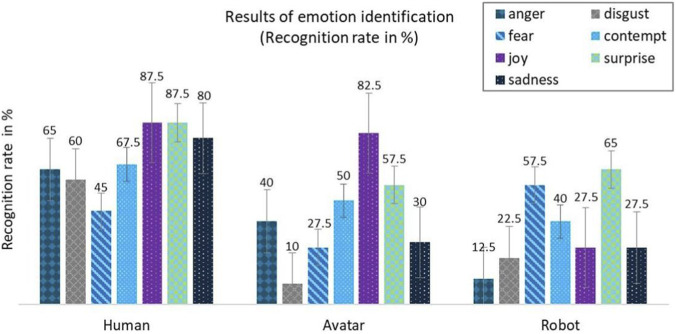
Result of the correct identification of emotions in %, n = 40.

Figure 5 shows the results of correct emotion recognition for 40 participants who assessed the basic emotions (anger, disgust, fear, contempt, joy, surprise, sadness) in three different representations: human, avatar, and humanoid robot. Table 2a (Supplementary Material) shows the individual recognition rates (n) of seven basic emotions when portrayed by humans, avatars, and robots. Overall, human emotions were recognized best, followed by robotic emotions, while avatar portrayals had the lowest identification rates:. The percentages represent the relative frequency of the number of correctly identified basic emotions in relation to the total number. Each bar shows the relative frequency of the respective correctly identified basic emotion. The emotions on the human face were most often identified correctly, while those on the avatar and robot were less accurate, to varying degrees.

Table 2 below shows the percentage differences in the recognition of different emotions when comparing human representation and avatar representation (ΔHA), human representation and robot representation (ΔHR), and robot representation and avatar representation (ΔAR). Compared to human representation, avatars (ΔHA) showed an average recognition rate that was 28% lower (SD = 16.88), while the difference between human and robotic representation (ΔHR) was even more pronounced at −34.57% (SD = 25.09). In contrast, the comparison between robotic and avatar-based representation (ΔAR) showed only a small average difference of −6.43% (SD = 28.17), indicating similar performance of these two forms of representation in emotion recognition.

**TABLE 2:** Difference detection rate between different emotions in percent.

Statistic	ΔHA (%)	ΔHR (%)	ΔAR (%)
M (SD)	−28 (16.88)	−34.57 (25.09)	−6.43 (28.17)

### 3.2 Differences in identification rates in comparison

#### 3.2.1 Human and avatar (Δ HA)

Differences between the recognition rates for humans and avatars (Δ HA); negative values mean that human facial expressions are recognized better than avatar facial expressions. Positive values mean that an emotion is identified more often as correct on the avatar than on the human. Particularly significant losses are evident in the identification of the emotions surprise (impaired recognition of −30%), sadness (−50%), and disgust (−50%). For the emotions of joy (−5%) and contempt (−17.5%), the recognition rate was only slightly worse. Recognition rates deteriorate across the board, indicating limited transferability of human facial expressions to avatars.

#### 3.2.2 Avatar and robot (Δ AR)

Here, the difference between avatar and robot representation becomes visible. Positive values mean that the robot’s facial expressions are recognized better than the avatar’s facial expressions. The recognition rate for the emotions surprise (+7.5%), fear (+30%), and disgust (+12.5%) is significantly higher for the robot than for the avatar in some cases. The emotions anger (−27.5%), contempt (−10%), joy (−55%) and sadness (−2.5%) are portrayed more realistically by the avatar; here, the recognition rate is higher for the avatar than for the robot. The differences in recognition between avatars and robots can be explained by the methods of representation - robots convey dynamic, expressive emotions better, while avatars depict subtle facial expressions more precisely. Recognition performance therefore depends on a combination of dynamism and attention to detail.

#### 3.2.3 Humans and robot (Δ HR)

The transfer from humans to robots also shows a reduction in recognition rates, but in some cases this reduction is slightly less than for avatars. The greatest losses occur with anger (−52.5%), sadness (−52.5%), and disgust (−37.5%). The smallest losses occur with fear (−27.5%) and joy (−60%). Emotions with strong, clear, and dynamic movement patterns (e.g., fear, surprise) appear to be easier to transfer to avatars and robots, as the characteristic features of facial expressions remain recognizable even in mechanical systems or simplified facial models. More subtle or ambivalent emotions (e.g., disgust, anger, contempt) involve minor changes in the face that can only be represented to a limited extent by robot faces or avatars. In addition, robots and avatars have a limited number of movable components, which means that certain muscle movements cannot be reproduced exactly. An important thing is that errors do not pile up during transmission. This is because human facial expressions are not passed on sequentially or recursively from one representation to the next. Instead, each emotion is mapped directly to a defined representation of the avatar or robot. Inaccuracies are thus limited to individual expression components and do not have a cumulative effect across multiple transmission steps.

### 3.3 Identification of basic emotions in an example scene

**FIGURE 6:**
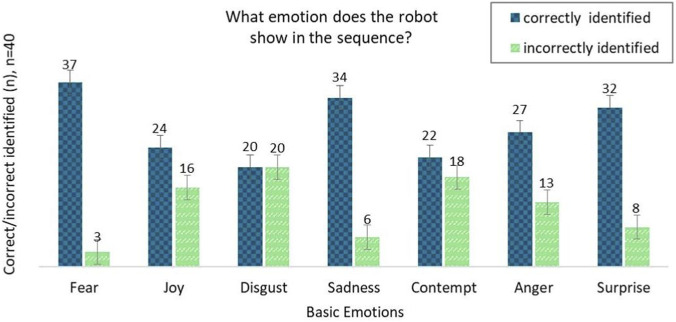
What emotion does the robot show in the animated sequence (n = 40)?.

In this study, 40 participants were shown short video sequences of a humanoid robot expressing one of seven basic emotions. After each sequence, the participants were asked to identify the emotion shown. The diagram in Figure 6 shows the number of correctly and incorrectly identified emotions. Supplementary Table 3a shows the number of correctly identified and incorrectly identified sequences.

The results show that most emotions are identified correctly, indicating that movement and context information through speech supports emotional perception. Fear, sadness and surprise achieved particularly high recognition rates. Anger and contempt were in the middle range, while joy and disgust had lower recognition rates.

### 3.4 Uncanny valley effect–robots and avatars in comparison

**FIGURE 7:**
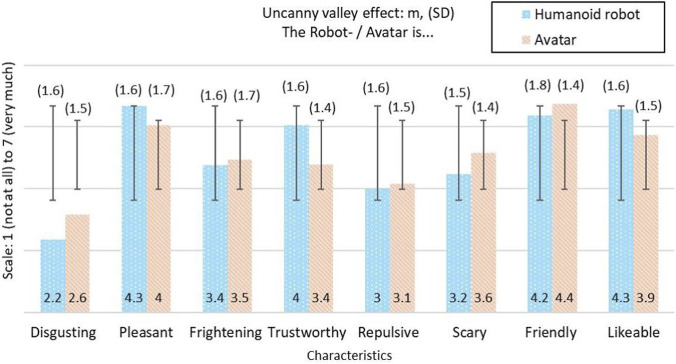
Mean values (m) and standard deviation (SD) for uncanny valley effect on humanoid robot and avatar (n = 40) on a Scale from 1 = not at all to 7 = very much.

Figure 7 shows the mean values of the survey on the uncanny valley effect based on the evaluation of eight different characteristics for robots and avatars. Supplementary Table 4a shows total values, mean values, and standard deviations. The robot is rated more positively than the avatar in almost all categories (except friendliness).

## 4 Discussion

### 4.1 Identification of basic emotions

Overall, it is clear that transferring human facial expressions to artificial systems leads to significant changes in recognition rates in some cases. In human representations, the emotions with the highest recognition rates were identified. This is particularly evident in joy, sadness and disgust. Surprise and anger also show comparatively high values. Only fear and contempt were less frequently recognized correctly.

Losses are usually recorded in both avatar and robot representations, although the extent varies depending on the specific emotion. A direct comparison between avatars and robots shows that the two systems have different strengths and weaknesses in emotional representation.

The average recognition rate decreases for avatars. The highest recognition rates are for joy and surprise, while fear and contempt were particularly poorly identified. This suggests that emotional nuances, especially negative or complex emotions, are perceived less clearly in virtual faces.

In the case of robot representations, recognition rates lie generally between those of avatars and humans, but show greater variation between emotions. Surprise and fear were recognized relatively well, while anger and disgust were rarely identified correctly.

The findings demonstrate a clear reduction in emotion recognition when human facial expressions are transferred to artificial representations. Compared to humans, recognition rates were markedly lower for avatars ΔHA (−28%) and robots ΔHR (−34.57%), while the difference between avatars and robots was relatively small ΔAR (−6.43%), indicating comparable overall performance. The largest impairments occurred for surprise, sadness, and disgust, which also showed significant statistical effects with medium to large effect sizes. In contrast, joy and contempt remained relatively stable across representations and did not differ significantly. These results suggest that subtle or complex facial cues are more difficult to reproduce in artificial systems, whereas more robust emotional expressions are less affected by representational changes.

Recognition rates decreased for both avatars and robots, but the pattern differed by representation type. Avatars showed a general reduction with strong impairments in surprise (−30%), sadness (−50%), and disgust (−-50%), whereas joy (−5%) and contempt (−17.5%) remained relatively stable. Robot representations also showed reduced recognition with greatest losses for anger (−52.5%), sadness (−52.5%), and disgust (−37.5%), while fear (−27.5%) and surprise (−30%) were comparatively less affected. These results support prior findings that negative and complex emotions are more prone to misrecognition (Dores et al., 2020; Shichuan and Martinez, 2011) and extend them to human–robot and human–avatar interactions. They highlight that artificial systems may reliably transmit broad, high-intensity expressions, but struggle with nuanced emotional cues, which is critical for applications in patient simulation and social robotics.

No correlation was found between demographics (age, gender, professional background) and recognition rates, suggesting that the observed effects are primarily due to representation rather than participant factors.

### 4.2 Identification of basic emotions in a dynamic scene

The results show that the majority of emotions were reliably recognized, suggesting that movement and contextual information especially in conjunction with speech -support emotional perception. Dynamic emotions such as fear, sadness, and surprise in particular showed high recognition rates. Emotions such as anger and contempt were in the middle range of recognizability, while joy and disgust were comparatively more frequently misinterpreted. This suggests that more subtle or ambivalent emotions, especially disgust, are more difficult to interpret in mechanically rendered facial expressions.

These findings address our second research question regarding the role of movement and context in emotion recognition and suggest that dynamic, multimodal representations (combining facial movement and speech) enhance recognizability, a critical consideration for socially interactive robots.

### 4.3 Uncanny valley effect–robots and avatars compared

The results indicate that humanoid robots are generally perceived more positively than avatars, particularly on trustworthiness, friendliness, and likeability, despite some lingering strangeness. This aligns with prior research suggesting that physical presence and subtle social cues contribute to social attractiveness and engagement, even when facial realism is limited. This tendency suggests that the physical presence and social signals of the robot can promote social attractiveness and willingness to interact despite a certain strangeness. Overall, the results indicate that the humanoid robot is perceived slightly more positively than the avatar in the conflict between familiarity and strangeness.

### 4.4 Limitations

The study has several limitations. The humanoid robot’s facial expressions were technically limited by the number of actuators and degrees of freedom, which meant that facial expressions could only be shown to a limited extent in some cases. There were also differences in the visual and expressive qualities of the images of the avatar and the robot, which affected the comparability of the results. Methodologically, the small sample size should be noted, which limits the generalizability of the study results. In addition, individual differences in emotion assignment and subjective evaluations may have distorted the results. Furthermore, this study worked exclusively with the seven basic emotions according to Ekman, while complex or mixed emotions and longer interaction sequences were not taken into account. Future studies should include larger samples, more realistic scenarios, and multimodal approaches in order to further optimize the emotional ability of humanoid robots.

### 4.5 Conclusion

The results of the study show that it is generally possible to transfer human facial expressions to humanoid robots, but that there are significant differences in the recognition rate between the individual emotions. Expressive emotions such as surprise and fear could be reproduced with high accuracy by the robot, while more subtle emotions such as anger or disgust showed lower agreement values. Technical limitations in the representation of fine muscle movements and the limited facial resolution of the actuators could be the reason for this. In addition, the results indicated that dynamic, animated facial expressions in combination with speech significantly improve emotional recognition, underscoring the importance of temporal and contextual factors for the interpretation of non-human facial expressions. Overall, the participants rated the humanoid robot more positively than the avatar, indicating a believable approximation of human expressions. Furthermore, the results highlight the potential of humanoid robots in patient simulation, particularly for simulating mental illnesses. Realistically reproduced emotions can be used to create interactive training scenarios that support therapeutic and diagnostic processes. The further development of fine motor facial components and the integration of dynamic expression models offer a promising approach to realistic simulation of social-emotional interactions.

## Data Availability

The raw data supporting the conclusions of this article will be made available by the authors, without undue reservation.
