# Supplementary material for: Automated capture and transfer of human facial expressions to humanoid robots for realistic patient simulation
Source: Front Robot AI. 2026 Mar 24;13:1798227. doi: 10.3389/frobt.2026.1798227 (PMC13054010; doi:10.3389/frobt.2026.1798227)
Supplement: Supplementary file 1 [file Supplementaryfile1.pdf]

Supplementary Material

Supplementary Table 1a. Assignment of the corresponding blendshape values (BV) to the robot's degrees of freedom (robot positions, RP)

**Robot positions RP**	**Blendshape Value (BV)**
mouth smile left	smile l
mouth smile right	smile r
jaw open	mouth open
jaw right/jaw left	jaw sleve
top eyelids L	eyeblink l
top eyelids R	eyeblink r
bottom eyelids L	eyeWide L /eyesquint
bottom eyelids R	eyeWide R /eyesquint
Brows L	brow down L
Brows R	brow down R
Outer Brows L	brow outer up L
Outer Brows R	brow outer up R

Supplementary Equations: Mathematical Formulation of the Transfer (Equations 1-6)

The following describes the mathematical conversion of blend shape values (BV) into robot positions (RP), which was implemented in Python and uses the tool Virtual Robot in a simulation-in-the-loop approach. From the 52 blend shape values captured from the human face using MediaPipe, selected points were assigned to the 13 robot actuators. The selection and identification of the corresponding blend shapes for the robot positions can be seen in the Supplementary Material, Table 1a. (Assignment of the corresponding blendshape values to the robot's degrees of freedom). The aim of this calculations is to translate human facial expressions into optimal and mechanically implementable movements of the robot actuators.

Step 1: The difference between human facial expressions (H) with a neutral facial expression (n) and facial expressions corresponding to an emotion (i) is calculated using the equation below via the blendshape values (BV) (Equation 1). The blendshape values were determined for the image of the human (H) with neutral facial expressions (n), as well as for the image with one of the seven basic emotions (i = 1...7) shown. Then, the difference (D) between the blend shape values for the neutral facial expression of the human and the facial expression for the corresponding basic emotion of the human was calculated for each of the 13 robot positions (j = 1...13) (step equation 1). x denotes the iteration index of the simulation-in-the-loop optimization. The difference $D_{i,j}^{x}$ describes the percentage deviation for the x'th iteration of the respective facial expression from the neutral facial expression and thus forms the basis for the transfer to the robot.

$$D^{x}_{i,j} = \frac{BV^{H}_{i,j} - BV^{H}_{n,j}}{BV^{H}_{n,j}} \qquad BV^{H}_{n,j} \neq 0 \qquad (1)$$

The differences determined were then transferred to the neutral facial expression of the humanoid robot by adjusting the values using the robot's blend shape values (BV^R). In accordance with the assignment of the blend shape values to the robot actuators (Supplementary Table 2a.), the selected keypoints could be transferred 1:1 to the corresponding facial position. In the process, it was checked whether the resulting values were within the defined degrees of freedom of the robot actuators (RP_{min} and RP_{max}). Values outside this range were limited to the next permissible limit to prevent overload or unrealistic movement of the actuator.

Step 2: A number of 13 robot positions (RP_j) can be set by the actuators ($j = 1...13$). For numerical stability, a virtual neutral value of 1 is used internally if $RP_{n,j} = 0$ which is compensated by subtracting 1 after scaling (Equation 2):

$$\mathrm{RP}^{x}_{\mathrm{i,j}} = \begin{cases} D^{x}_{i,j}, & (RP_{n,j} = 0) \\ RP_{n,j} * \left(1 + D^{x}_{i,j}\right), & (RP_{n,j} \neq 0) \end{cases} \qquad (2)$$

Step 3: The calculated values for the robot positions ($RP_{i,j}$) are checked according to the degrees of freedom and corrected if necessary (Equation 3):

$$\mathrm{RP}^{x}_{\mathrm{i,j}} = \begin{cases} RP_{min,j}, & RP^{x}_{i,j} < RP_{min,j} \\ RP_{max,j}, & RP^{x}_{i,j} > RP_{max,j} \end{cases} \qquad (3)$$

The theoretical target facial expression (g) for the respective emotion (i) of the robot is calculated using the blendshape values (step 4). After executing the robot positions for the emotion (i), new blendshape values are recorded for the result of the x'th + 1 iteration. In an iterative adjustment process, the generated blendshape values are compared with the calculated target values of human facial expressions.

Step 4: Based on the calculated difference, the theoretical target facial expression (g) of the robot is calculated using the blend shape values. The blend shape values of the neutral robot state ($\mathrm{BV}^{\mathrm{R}}_{\mathrm{n}}$) are multiplied by the difference (Equation 4). The result ($gBV^{R}_{i,j}$) describes the theoretically achievable blend shape values (g).

$$gBV^{R}_{i,j} = BV^{R}_{n,j} + \left(D_{i,j} * BV^{R}_{n,j}\right) \qquad (4)$$

Step 5: After executing the robot movement and remeasuring BV^R, the deviation between the theoretically achievable (g) and the actual blend shape values generated by the robot is checked for validation. The difference shows how much the actual simulation result deviates from the theoretical target facial expression in the respective run (Equation 5):

$$\Delta BV^{R,x}_{i,j} = \left| gBV^{R}_{i,j} - BV^{R,x}_{i,j} \right| \qquad (5)$$

This comparison is repeated iteratively in several iterations (x), with the robot positions (j) being adjusted step by step. The iterative optimization process was repeated until the deviation between the theoretically calculated and the actually generated blendshape values was ≤ 20%.

Step 6: In an iterative adjustment process, the robot positions are executed multiple times, the BV^{R} is recorded, and the robot positions are adjusted iteratively. In each iteration ($x = 1 \ldots N$), the result image of the robot generated in the first iteration is captured again by the facemesh, thus generating new $BV^{R,x}$. The iterative adjustment is repeated until the deviation falls below a defined value $D_{opt,i,j}$ (Equation 6):

$$D_{opt,i,j}^{x} = \frac{\left| gBV_{i,j}^{R,} - BV_{i,j}^{R,x} \right|}{gBV_{i,j}^{R,}} \leq 0.2 \qquad (6)$$

Table 3a. Statistics - identification rate of each basic emotions represented by human, avatar and robot

Emotions	correctly identified (n)	(%)	incorrectly identified (n)	(%)
**Human**				
Anger	23	57.5	17	42.5
Disgust	20	50	20	50
Fear	17	42.5	23	57.5
Contempt	23	57.5	17	42.5
Joy	36	90	4	10
Surprise	33	82.5	7	17.5
Sadness	29	72.5	11	27.5
**Avatar**				
Anger	15	37.5	25	62.5
Disgust	2	5	38	95
Fear	9	22.5	31	77.5
Contempt	18	45	22	55
Joy	31	77.5	9	22.5
Surprise	1	2.5	39	97.5
Sadness	9	22.5	31	77.5
**Robot**				
Anger	4	10	36	90
Disgust	7	17.5	33	82.5
Fear	22	55	18	45
Contempt	14	35	26	65
Joy	30	75	10	25
Surprise	24	60	16	40
Sadness	11	27.5	29	72.5

Table 3a. Emotion the robot shows in the animated sequence (Statistics)

	**Correctly identified**		**Incorrectly identified**	
	n	%	n	%
Anger	27	67.5	13	32.5
Disgust	20	50	20	50
Fear	37	92.5	3	7.5
Contempt	22	55	18	45
Joy	24	60	16	40
Surprise	32	80	8	20
Sadness	34	85	6	15

Table 4a. Statistic for uncanny valley effect on humanoid robot and avatar (n=40), standard deviation (SD) and mean value (m)

	**Robot**			**Avatar**		
Emotion:	Total (n)	SD	m	Total (n)	SD	m
Disgusting	83	1.60	2.13	95	1.48	5.00
Pleasant	169	1.57	4.33	148	1.66	7.79
Frightening	129	1.58	3.39	131	1.82	6.89
Trustworthy	157	1.61	4.13	131	1.65	6.89
Repulsive	112	1.59	2.95	117	1.48	6.16
Scary	120	1.44	3.16	135	1.44	7.11
Friendly	162	1.86	4.26	157	1.36	8.26
Likeable	166	1.61	4.37	137	1.49	7.21

Acting script (5a)

The actors were asked to act out the following sentences with corresponding facial expressions.

Joy: Example: "I can hardly believe I got the job—it feels like everything finally feels right!"

Sadness: Example: "It's hard for me to think that you won't be here much longer."

Fear: Example: "I don't know if I can handle this—what if I really fail?"

Anger: Example: "Why do I always have to put up with so much while everyone else just gets away with it?"

Surprise: Example: "I really didn't see that coming—I'm completely stunned!"

Disgust: Example: "The smell in here is almost unbearable—I can't believe anyone puts up with this."

Contempt: Example: "It's hard to understand how someone can do something like that without considering the consequences."

Questionnaire (7a): Measurement of the "Uncanny Valley Effect"

Please rate the robot/avatar on a scale of 1 to 7.

	1	2	3	4	5	6	7
Disgusting							
Pleasant							
Frightening							
Trustworthy							
Repulsive							
Scary							
Friendly							
Likeable							
